# The Molecular Phylogenetic Signature of Clades in Decline

**DOI:** 10.1371/journal.pone.0025780

**Published:** 2011-10-04

**Authors:** Tiago B. Quental, Charles R. Marshall

**Affiliations:** 1 Departamento de Ecologia, Universidade Estadual de São Paulo (USP), São Paulo, São Paulo, Brazil; 2 Museum of Paleontology and Department of Integrative Biology, University of California, Berkeley, California, United States of America; Institut de Biologia Evolutiva - Universitat Pompeu Fabra, Spain

## Abstract

Molecular phylogenies have been used to study the diversification of many clades. However, current methods for inferring diversification dynamics from molecular phylogenies ignore the possibility that clades may be decreasing in diversity, despite the fact that the fossil record shows this to be the case for many groups. Here we investigate the molecular phylogenetic signature of decreasing diversity using the most widely used statistic for inferring diversity dynamics from molecular phylogenies, the γ statistic. We show that if a clade is in decline its molecular phylogeny may show evidence of the decrease in the diversification rate that occurred between its diversification and decline phases. The ability to detect the change in diversification rate depends largely on the ratio of the speciation rates of the diversification and decline phases, the higher the ratio the stronger the signal of the change in diversification rate. Consequently, molecular phylogenies of clades in relative rapid decline do not carry a signature of their decreasing diversification. Further, the signal of the change in diversification rate, if present, declines as the diversity drop. Unfortunately, the molecular signature of clades in decline is the same as the signature produced by diversity dependent diversification. Given this similarity, and the inability of current methods to detect declining diversity, it is likely that some of the extant clades that show a decrease in diversification rate, currently interpreted as evidence for diversity dependent diversification, are in fact in decline. Unless methods can be developed that can discriminate between the different modes of diversification, specifically diversity dependent diversification and declining diversity, we will need the fossil record, or data from some other source, to distinguish between these very different diversity trajectories.

## Introduction

Understanding the controls of biodiversity is one of the main goals of ecology and evolutionary biology. In recent decades this endeavor has been revitalized by the use of molecular phylogenies to study the diversification of many clades [1]–[6]. Key has been the development of analytic methods for estimating speciation and extinction rates from molecular phylogenies, despite the absence of extinct species [7]–[13], and ways of investigating the tempo of diversification [1], [12], [14]. This is especially important given that many clades do not have a fossil record of sufficient quality (or any fossil record at all for that matter) to enable detailed diversification studies.

Currently, these tools have been used to distinguish between clades that are diversifying exponentially from those that might be undergoing diversity dependent diversification, and are at or approaching an equilibrium carrying capacity [4], [5], [14], [15]. In some cases the relative contributions of changes in speciation and extinction rate to overall diversification patterns have also been estimated [4], [14], [15]. Even though caution is warranted – over-dispersed sampling commonly used by biologists [1], [16] and under-parameterization of the DNA models [17] might mask true diversification patterns – many phylogenies show a pattern of decreasing diversification rates, which in turn are typically attributed to diversity dependent diversification [4], [5], [15].

However, molecular phylogenies, by the virtue of only considering extant species, carry the perceptual bias of increasing diversity [13], and most biologist, perhaps for this reason, work with the premise of expanding diversity with time, either unbounded (exponential growth) or with some sort of diversity saturation. Yet, the fossil record shows that many clades have been in decline for a significant part of their history, and, of course, many are now extinct [18]–[21]. Hence it is probable that many extant clades are also currently in decline, and have thus experienced negative diversification rates over their recent history. Clades for which the fossil record shows this to be true include the Cetacea [18], perissodactyl mammals, lungfish, brachiopods, stenolaemate bryozoans, gymnosperms, sphenophytes (the horse tails), etc [21].

Unfortunately, none of the current methods used for deducing diversity trajectories from molecular phylogenies incorporate this very real possibility of negative diversification rates [18]. Hence, if we want to understand whether molecular phylogenies are able to properly reveal a clade's true diversity dynamics, we need to understand what effect declining diversity will have on the appearance of molecular phylogenies. Here we use computer simulation to conduct this investigation, and explore the robustness of some of the ecological interpretations currently drawn from the analysis of molecular phylogenies, including testing the hypothesis that molecular phylogenies of clades in decline look similar to those driven by diversity-dependent diversification [18].

## Methods

### Simulation scenarios

The fossil record clearly shows that clades rise and fall in diversity [19]–[21] so our main goal is to use computer simulation to investigate the consequences of declining diversity on the diversification signature of molecular phylogenies. The rise and fall of clades could in theory happen in many different ways. For example, a clade could spend most of its “life” either in the rise phase, or in the decline phase, or have approximately symmetric rise and decline phases. Additionally, during either the rise or decline phases, the rates of extinction and speciation could be constant (i.e., exponential growth or decline) or decrease (or increase) with respect to time or diversity. There are obviously even more complex dynamics, but our purpose is not to explore all the possibilities, but to focus on a few simple scenarios to explore the first order molecular signature of clades in decline.

**Figure 1:**
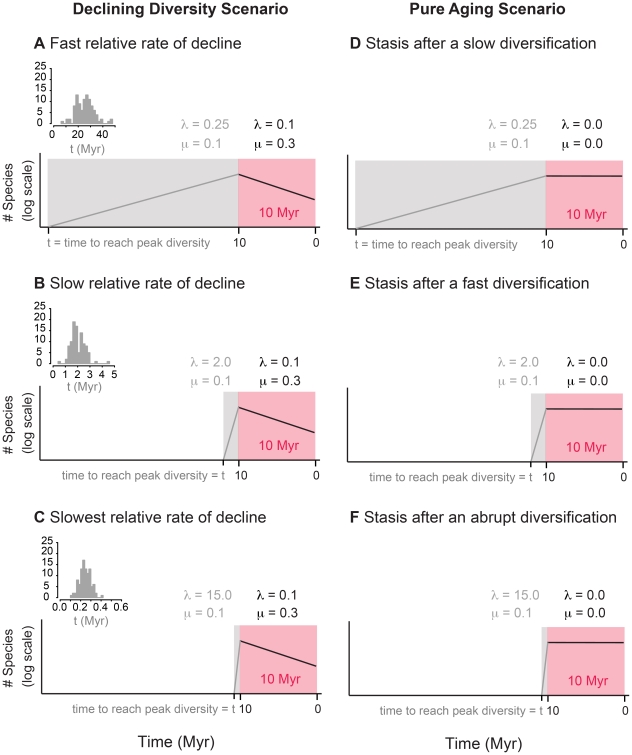
Cartoon of the different simulation scenarios employed in this paper. Panels A, B and C represent the clades in decline scenario, where the number of species rise (grey portion) and decline (pink portion) exponentially. Panels D, E and F represent the “stasis” scenario, where the number of species grows (grey portion) in a manner similar to the clades in decline scenario (A, B, and C) but then stays fixed thereafter (pink portion). The only difference among the simulations within each scenario is the speciation rate in the rising phase. This meant that each scenario took a different time to reach the peak diversity. The small histograms (top left of panels A, B and C) represent the time it took to reach the peak diversity for each scenario. Each simulation was run so that the final diversity was 10 lineages and the decline phase was set to last 10 million years. Given our simulation scheme, each group of simulations resulted in similar average peak diversities, with a mean of 76. 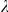 represents the speciation rate and 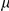 represents the extinction rate.

The specific simulation approach used here was motivated by importance of diversity dependent diversification reported in the literature [4], [5], [15], and our suspicion that exponential decline could leave a similar signature on molecular phylogenies [18]. Thus, we choose the simplest diversification scenario that would allow us to: 1) broadly characterize the molecular signature of clades in decline; 2) highlight potential shortcomings in current interpretations of the diversity dependent dynamics; and, 3) enhance our understanding of the extent to which molecular phylogenies can, or cannot, be used to study the diversification process. Thus, we simulated the diversity trajectories with an expansion of diversity at a constant rate followed by declining diversity, also at a constant rate (Figure 1).

For each set of simulations a total of 100 replicates were run, a sample size large enough to capture the stochastic variation in the models used. We used the R package TreeSim [22], [23] for simulating trees and the R packages Geiger [24] and paleoPhylo [25] to manipulate and analyze the data. TreeSim enabled us to run simulations that conditioned both on a specific final diversity (10 lineages) and on a specific duration for the decline (10 million years).

### Metric for comparing simulated phylogenies

Several different tools are available for using molecular phylogenies to infer diversity dynamics [1], [7]–[9], [12], [14]. However, it is becoming apparent that the methods developed for estimating speciation and extinction rates are unable to estimate extinction rates when clades are in decline [18], which in turn leads to inaccurate estimates of speciation rates as well [18]. Given the inability of these methods to handle clades in decline, we chose to work with a simpler method, the γ statistic [1], introduced to simply detect changes in the diversification rate. We note, however, that while the γ statistic cannot be used to estimate speciation and origination rates, per se, simulation studies show that when the γ statistic indicates a change in the diversification rate, it must have been driven in part by a decrease in the speciation rate [15], [18], [26].

The γ statistic has some other advantages. First, it is easily understood (negative values mean the nodes are concentrated deep in the tree). Second, it is widely used, especially in the literature on diversity-dependent diversification [1], [4]–[6], [18]. Third, by virtue of being a summary statistic, it allows straightforward comparison among trees (although see below for a discussion of its dependence on tree size).

The γ statistic does have its limitations, primarily an appreciable type II error rate. Specifically, it has low discriminating power between exponential growth and the early phases of diversity dependent diversification [27], or when the diversity dependent diversification is driven by a low initial speciation rate relative to the equilibrium extinction rate [26], or if clades are experiencing species turnover at an equilibrium diversity [27]. Similarly, it has low discriminating power when one is trying to distinguish between subtly different models of diversification [14] (although here we are not trying to distinguish between subtly different models of diversification). Second, as a summary statistic, it may not handle cases where the diversification dynamics change appreciably through the history of a clade (for example, late bursts of speciation can mask early bursts of diversification [28]. However, it has a negligible Type I error rate – an inference of decreasing diversification rate appears to be a reliable inference.

### Clades in decline

The rise and fall of simulated clades was modeled by exponential growth followed by exponential decline. We chose this way of simulating the waxing and waning of diversity because preliminary data suggested that the resulting phylogenies would give the appearance of diversity dependence, and so we wanted to make sure that we did not confound the interpretation of our results by having the initial diversification process be diversity dependent. We chose a range of realistic speciation and extinction rates [21], although the absolute values are not relevant if one is interested in investigating the topologies of molecular phylogenies under different diversification scenarios [26], [27]. Figure 1A–C show the rates used. Figure 1A (fast relative rate of decline) illustrates a scenario where the loss of diversity is fast compared with the rate of accumulating diversity. The scenarios shown in Figure 1B (slow relative rate of decline) and 1C (slowest relative rate of decline) represent scenarios where the rate of loss of diversity is progressively slower than the initial rate of accumulation of diversity. For ease of comparison, in all simulations the decline phase was modeled with the same constant extinction and speciation rates, and the extinction rate during the initial diversification was also held constant. We also arbitrarily chose to condition the decline phase to a time span of 10 million years and a final diversity of 10 species, although the specific values chosen do not affect our main conclusions (data not shown; see also [26]). This conditioning meant that the average peak diversity was the same among different scenarios, but that the average time taken to reach that diversity varied among the three decline scenarios (see histogram in upper left of panels A, B, and C in Figure 1).

### Analyzing the simulations – time traveling

**Figure 2:**
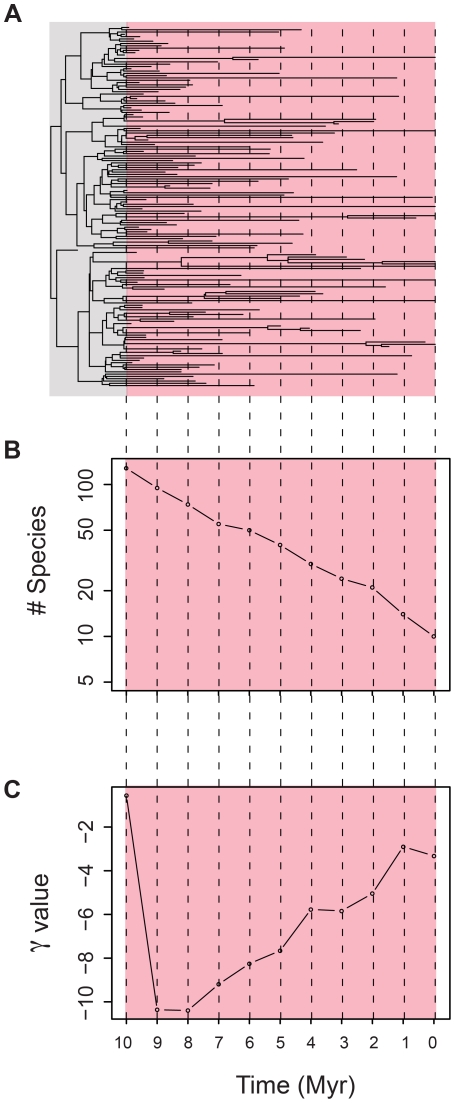
An exemplar simulation of a clade in decline. Rates of increase and decline used correspond to the slow relative rate of decline from Figure 1B. A) Exemplar phylogeny. The pink portion represents the decline in diversity phase. The dashed lines represent points in time where the γ value and diversity of each phylogeny was assessed. B) The number of extant species at each point in time for decline phase. C) The γ statistic for what would have been the molecular phylogeny at each point in time for the decline phase.

To characterize the molecular signature of the declining diversity as it unfolds, we calculated the γ statistic at different points in time, a procedure we term “time traveling” (see Figure 2 for an outline of the procedure; see also [27]). The molecular signature of the diversification phase of our simulations is the well-understood constant birth-death process [7]–[9], [11], and was not examined further here. Figure 2A shows an exemplar phylogeny, with extant and now-extinct taxa. The pink box highlights the decline phase. We “time traveled” back in time every 1 million years (represented by the dashed lines) from the present to the time when diversity peak was reached, 10 million years before the present. At each point in time we calculated the number of species extant and the γ statistic for what would have been the molecular phylogeny (only the extant species) at that point in time (see Figures 2B and 2C). In some cases the γ statistic changed so fast that additional time points were analyzed to capture its behavior as diversity was lost.

The reason time-traveling analysis is required is that in the absence of a good fossil record it is virtually impossible to know, even if you knew your clade was in decline, how long ago peak diversity was reached, and what its peak diversity was [18], [27]. Thus for a given phylogeny, we don't typically know where it is in its “ontogeny” – time traveling is needed if one wants to investigate how well molecular phylogenies store a record of their diversification history at different points in their history.

## Results

### The signature of declining diversity

**Figure 3:**
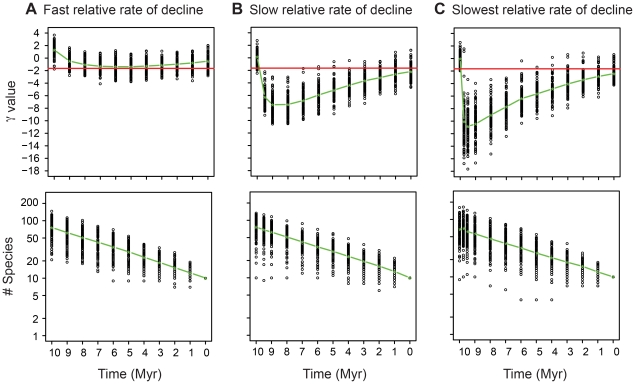
The γ statistic and number of species through time for all simulated trees for the decline phase (last 10 MY) for the clades in decline simulations (**Figure 1A–C**). Note that while the diversity declines are effectively the same for all three simulations (bottom row), the different diversification phases result in different γ values (upper row). The green line represents the average γ statistic or average number of species. The red line represents the 5% cutoff point for rejecting the null hypothesis of constant diversification (γ = −1.645).

Our simulations show that clades in the initial phases of their decline typically result in molecular phylogenies with the most negative γ values (Figure 3A–C). The strength of the signal of the decline depends, at least in part, on the magnitude of the ratio of the rate of speciation in the diversification (waxing) phase to the rate of speciation in the decline (waning) phase, 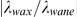. The higher the ratio (the lower the relative rate of decline), the more negative the γ statistic (Figure 3A–C). When the ratio is too low (i.e., when the relative rate of decline is high), for example when 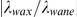 is 2.5 (Figures 1A and 3A), the null hypothesis of a constant diversification rate will not usually be rejected (the resulting γ values are only rarely <−1.645). In this case the exponential decline would most likely be interpreted as exponential diversification, as appears to have happened in a molecular phylogenetic analysis of the diversity dynamics of the living cetaceans [18], [29], although as we note above, the γ statistic has low statistical power – a γ value>−1.645 could also mean the early phases of diversity dependent growth [27]; diversity dependent growth with low a ratio between the initial speciation rate and equilibrium extinction rate [26]; or, species turn-over at equilibrium diversity [27]. Given that molecular phylogenies only directly store information of cladogenic events it is perhaps not surprising that if the change in speciation rate is not high enough there will not be enough power to detect the change in the diversification rate.

When 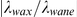 is high (the relative rate of decline is slow), for example 20 (Figures 1B and 3B), in most cases the negative diversification rate is reflected in significantly negative γ values. In addition, the higher the ratio, the more quickly in time the γ values become significantly negative, and the longer the γ value stays significantly negative (Figure 3). For example, in our simulations when the ratio is low (Figure 3A) the most negative values of γ are only seen several million years after the onset of the decline, while at higher ratios (Figure 3B, C) the most negative γ values are seen within a million years of the onset of the decline, and γ stays significantly negative until the clade is nearly extinguished.

### Meaning of the negative γ values

Negative γ values are generally taken to mean that there was a decreasing diversification rate, and more specifically a decrease in the speciation rate [4], [26]. Our simulations are consistent with this observation – in each simulation there was a large and instantaneous drop in the diversification rate as we switched from the waxing phase to the waning phase, which we achieved largely through a decrease in the speciation rate (see Figure 1A–C). Thus, while negative γ values have been traditionally used as evidence of diversity dependent diversification [4], [5], [14], [15], in our simulations there is no diversity dependence. Thus, these simulations show that there is more than one way (in an evolutionary/ecological sense) to generate negative γ values (see discussion below).

### Why does declining diversity lead to negative γ values?

Our basic finding is that the γ values drop as soon as the diversification rate becomes negative, and that the γ values can be surprisingly negative. However, as the diversity continues to drop, the γ value rises again, and can ultimately become positive again mimicking exponential growth (among other scenarios – see above), when in fact the clade is in exponential decline. We wished to understand why the γ values become negative in the first place, and why γ then becomes more positive as the clades continue to decline in diversity, and so we ran additional simulations to explore these questions.

### Effects of under-sampling after exponential growth

Under-sampling species will generate a predictable bias in the diversification signature of molecular phylogenies by artificially increasing the relative importance of early splitting events [1], [16], which will lead to more negative γ values. Following this observation, we [18] suggested that extinction could be viewed as evolutionary under-sampling (the failure to sample species due to extinction, rather simply failing to collect them in the field), which led us to posit that clades in decline might also have negative γ values.

To test this supposition, we ran a set of simulations with simple exponential growth followed by different degrees of under-sampling after a pre-assigned fixed diversity had been achieved. For these simulations we used the same speciation and extinction rates used in the initial diversification phase of our clades-in-decline simulations (Figure 1). For the under-sampling simulations the final diversity was set to 76 (the average peak diversity of the clades-in-decline simulations) and 760 species to explore the effect of the absolute number of species on the signal introduced by under-sampling of the terminal branches. The simulated trees were progressively under-sampled until only 10 species remained, the final diversity in our clades-in-decline simulations. Terminals were removed at random.

**Figure 4:**
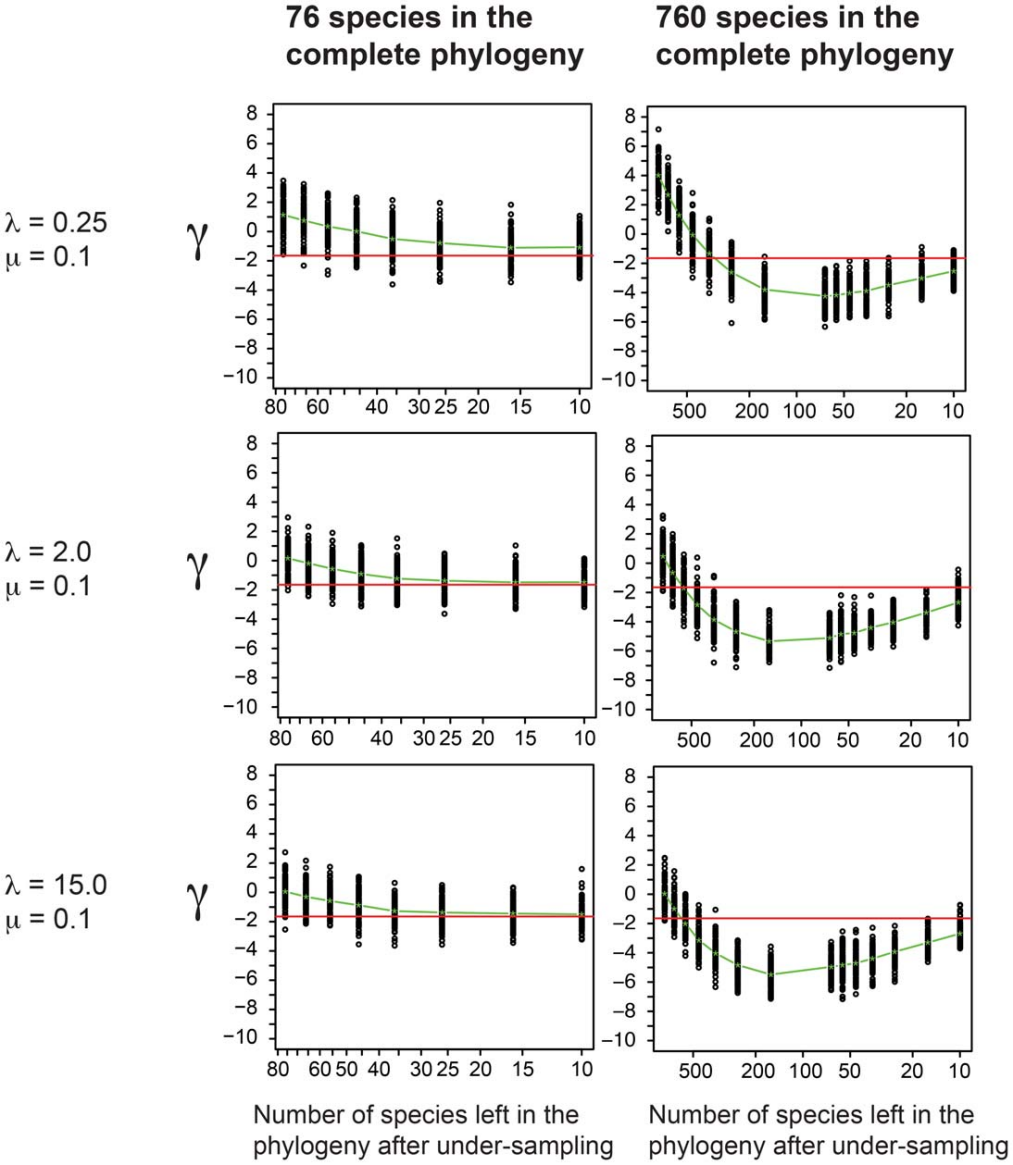
The effects of under-sampling on the γ statistic. The phylogenies were generated using the same three exponential rates of increase used in the clades-in-decline simulations (grey portion of Figures 1A–C). Two final diversities were used, 76 species (left column) and 760 species (right column). The green line represents the average γ statistic. The red line represents the 5% cutoff point for rejecting the null hypothesis of constant diversification (γ = −1.645). Under-sampling has a surprisingly small effect on the γ value – the reason for the highly negative γ values seen in our simulations has little to do with extinction mimicking the effects of under-sampling.

While under-sampling made the γ statistic more negative than the fully sampled tree, γ only became significantly negative for the 760 species scenario (Figure 4) – under sampling via extinction is not the primary driver of the negative γ values found in our simulations of clades in decline, having only a minor effect on the γ value.

### Clades in stasis – the effects of aging

Given that the loss of terminals, per se, is not responsible for the strongly negative γ values seen in our simulations of clades in decline, what could be responsible? Our second guess was based on the realization that one way to achieve strongly negative γ values is to simply extend the lengths of the terminal branches, without adding or subtracting branches. Thus, we ran a set of “pure-aging” simulations, initiated with exponential growth with the same rates of initial diversification used in the clades-in-decline simulations (Figure 1A–C). Then, instead of switching to exponential decline, the branch lengths were simply extended for 10 million years (see Figure 1D–F). Two peak diversities were used: 10 and 76 species. These values were chosen because in our simulations of clades in decline the average peak diversity was 76 species (see above) and because we conditioned on a final diversity of 10.

**Figure 5:**
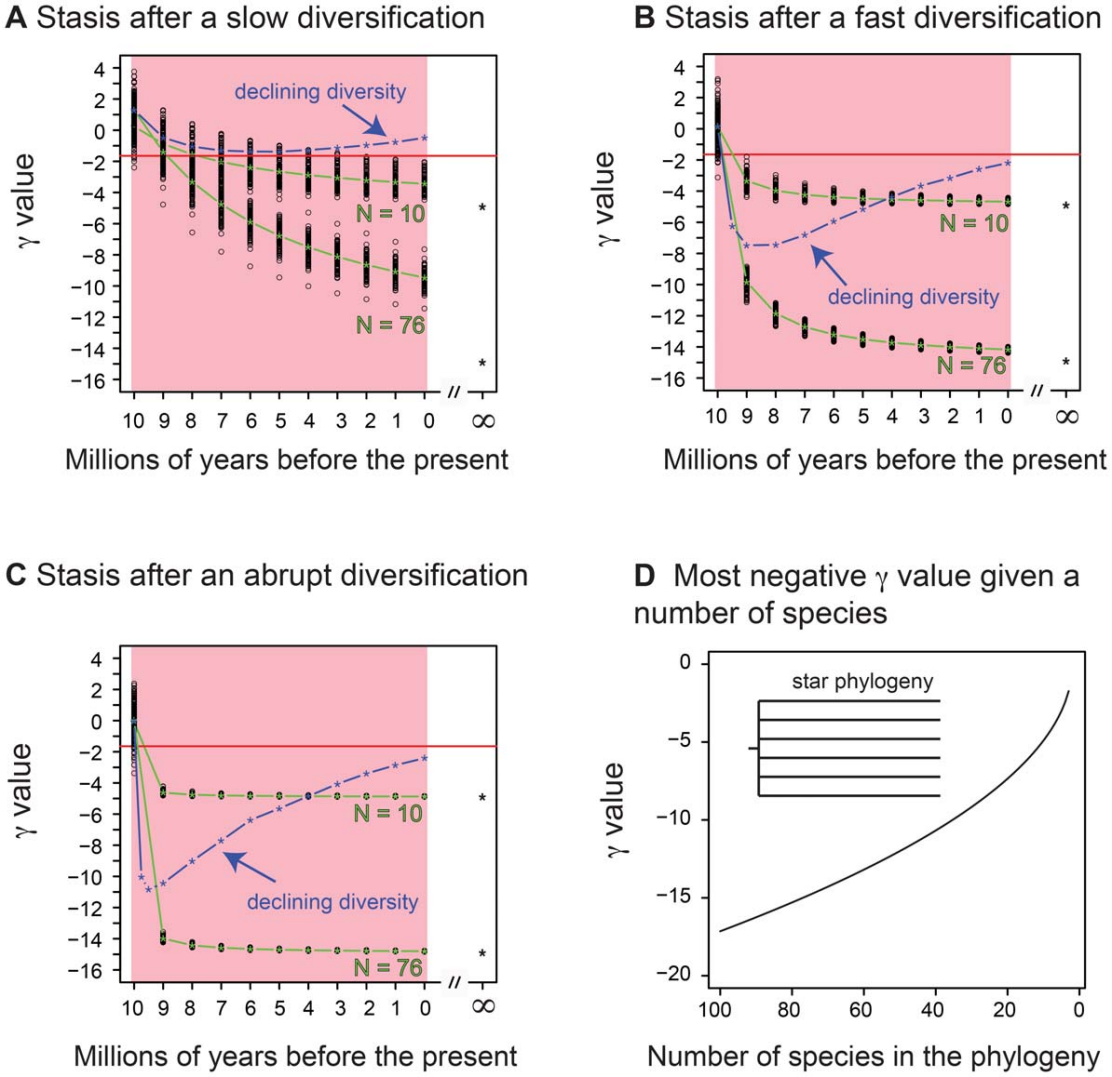
A–C) The evolution of the γ statistic (green line) for all simulated trees in the pure aging scenario after initial exponential growth. A) Stasis after a slow diversification. B) Stasis after a fast diversification. C) Stasis after an abrupt diversification. The asterisk at time = ∞ represents the most negative value possible for the γ statistic for a given phylogeny with 10 or 76 species, which corresponds to a phylogeny after an infinitely long aging phase (i.e., a star phylogeny). The blue line represents the average γ statistic for the clades in decline simulations (data from Figure 3). The red line represents the 5% cutoff point for rejecting the null hypothesis of constant diversification (γ = −1.645). D) The expected most negative value for the γ statistic for given phylogeny as a function of the number of species (based on [6]); this represents a phylogeny with a star topology.

As expected, the clades in stasis produce highly negative γ values (the green lines in Figure 5A–C). As time passes the γ value continues to decline, and after an infinite amount of time the γ value will reach the maximum possible negative value given the number of species in the phylogeny (the “star phylogeny” scenario described by McPeek [6]; Figure 5D). For the simulations with relatively rapid diversification phases (Figure 1B,C) after 10 Myr the γ values of our simulated trees under the stasis scenario are indeed very close to this most negative potential value (Figure 5B,C).

However, Figure 5 shows that the simulated data for clades in decline never produce γ values as negative as seen in the pure aging scenarios. There are two reasons for this. The first and most important is that the maximum γ value is a function of the number of terminal branches ([6]; Figure 5D). In the clades in decline simulations, while on average the simulations start with 76 species, that number steadily drops as the extinction proceeds to a final diversity of 10 lineages. Thus we expect the γ value to become less negative as the extinction proceeds. However, if this were the only factor at play, then the simulations of clades in decline should have γ values that lie between the pure-aging scenarios for a diversity of 76 terminals and 10 terminals. However, the γ values continue to rise beyond the values expected for a star phylogeny of 10 species (see Figure 5). The reason is that in our simulations while there is a net loss in diversity, we employed a positive speciation rate, and so new nodes are also being added, making the γ values more positive. The validity of this reasoning was confirmed when we re-ran the simulations for the “stasis-after-fast-diversification” scenario (Figure 1E), with no speciation during the decline phase – the γ values lie, as expected, between the pure-aging scenarios with the diversities of 76 terminals and 10 terminals (see Supplemental Figure S1).

## Discussion

### The molecular signature of declining diversity

Our results clearly show that molecular phylogenies of clades experiencing a decline in diversity will present a molecular signature of the decreasing diversification rate, the switch from the waxing phase to the waning phase. Consistent with previous studies that show that changes in speciation rate are more important than changes in extinction rate on the appearance of molecular phylogenies [4], [18], we find that the ratio of the rate of initial speciation to the rate of speciation during the decline plays a major role in determining whether the molecular phylogeny will exhibit the signature of the switch in diversification rate (Figure 3). As noted above, given that molecular phylogenies only directly store information of (some) cladogenic events, and not the extinction events, it is perhaps not surprising that if there is insufficient change in the speciation rate between the waxing and waning phases that there will not be enough power to detect the changes in the diversification rates. There are other factors, such as the time spent in each phase (which is determined by the diversification rates in each phase) and the ratio of the extinction rates in the two phases that influence the exact signature of decreasing diversification as well. However, unlike diversity dependent diversification where a simple metric (the LiMe ratio of Quental and Marshall [26], the ratio of the initial speciation rate to equilibrium extinct rate) controls the molecular signature of the diversification scenario, we have been unable to find a simple metric that fully captures the behavior of the γ statistic for clades in decline.

Our initial hypothesis that the elevated extinction rate acts as the evolutionary equivalent of under-sampling [18], while technically true, is not the primary reason declining diversity leads to strongly negative γ values. The primary reason for the strongly negative γ values is the drop in the speciation rate that results in the relative absence of new nodes, coupled with the passage of time that extends all the branches that have so far escaped extinction – once the diversification rate becomes negative the aging of the surviving lineages results in the vast majority of nodes becoming concentrated deeper and deeper in the tree. And then, as the diversity continues to drop, the γ values begin to rise, largely due to the fact that γ is a function of total diversity of the phylogeny (Figure 5D), but also because the low levels of speciation in the decline phase continue to add some young nodes to the phylogeny. In some cases, the effect of decline-phase speciation can cause the γ values to become positive again, especially when the decline has persisted for long enough that most of the original speciation events have been lost to the molecular phylogeny due to the ongoing extinctions.

The dependency of γ on the number of taxa, also noted by McPeek [6], can be alleviated by normalizing all gamma values by the maximum possible value for the number of taxa present, but we do not pursue this further here. However, we note that when using the γ statistic to test against the null hypothesis of a constant birth-death process, the original intention of the γ statistic [1], the critical value for rejecting the null hypothesis is not dependent on the number of species. The distribution of gamma values will always be centered on zero and with a 5% cutoff at −1.645 for a pure birth process regardless of the total diversity (because exponential growth is self-similar), or shifted towards more positive values when extinction is present, which simply increases the type II error [1]. Thus, the dependency of γ on the number of taxa seen in our simulations does not undermine its original use as a simple and effective way of testing the null hypothesis of constant rates of diversification, but it does indicate, as noted above, that it has lower power if used to discriminate between a wide variety of diversification processes (see also [18]).

### The meaning of a decrease in diversification rate

Analyses of a large number of molecular phylogenies have repeatedly suggested the prevalence of decreases in diversification rates [4], [5], [14], [15]. Apart from the potential artifacts – non-random sampling [1], [16] and under-parameterization of DNA models [17] these results have been traditionally interpreted as evidence for diversity dependent diversification [4], [5], [14], [15]. Even though the field has seen some promising methodological advances [14], we suspect that this conclusion is premature given the limitation of the methods used [18], [27], [30] and the fact that none of them incorporate the possibility of declining diversity (but see [9]). In this context, our results show that a clade in decline can produce a molecular phylogeny with a signature of decrease in diversification rate (when viewed through the γ statistic), but that the decrease in diversification rate results from the switch from exponential growth to exponential decline, rather than due to diversity saturation, the standard interpretation.

Given our results, and the frequency with which declining diversity is found in the fossil record [19]–[21], we suspect that the high frequency of molecular phylogenies that show decreasing diversification rates [5], [6] is due to some of these clades being in decline, rather than because they are all undergoing diversity dependent diversification, a conclusion supported by a recent analysis of a snake molecular phylogeny [31]. In fact, the eroding effect of extinction, and the observation that diversity-dependent diversification with low initial speciation rates may frequently escape detection [4], [27], led Quental and Marshall [18] to suggest that there is in fact too much evidence of diversity dependent diversification, and thus that there are probably other mechanism(s) responsible for the large number of phylogenies that show decreasing diversification rates. Our results here confirm that suspicion, and provide an alternative diversification model that will lead to molecular phylogenies that might be interpreted as supporting diversity-dependent diversification. Indeed, while the observation that a molecular phylogeny has a decreasing rate of diversification, once made, appears robust, it also appears that many different diversification processes can produce very similar molecular phylogenies, making it almost impossible to determine the true process of diversification without the help of independent evidence, such as the fossil record (see also [18]). Given current methodological limitations and the absence of sufficient fossils for many clades to make inferences about past diversity trajectories, one of our highest priorities must be the development of methods for discriminating between all the possible mechanisms that can lead to decreasing rates of diversification in molecular phylogenies, including the likelihood of declining diversity.

## Supporting Information

Figure S1 The γ statistic through time for a decline diversity scenario without any speciation in the decline phase (last 10 MY). Rates of speciation and extinction used here were chosen to produce the same diversification rates in the rise (speciation = 2.0; extinction = 0.1; r = 1.9) and decline (speciation = 0.0; extinction = 0.2; r = −0.2) phases as used in the scenario shown in figure 1B. The blue line represents the average γ statistic. The red line represents the 5% cutoff point for rejecting the null hypothesis of constant diversification (γ = −1.645). The green lines represent the average γ statistic for simulated trees in the pure aging scenario after initial exponential growth for a peak diversity of 10 and 76 species (same as in figure 5B). The asterisk at time = ∞ represents the most negative value possible for the γ statistic for a given phylogeny with 10 or 76 species, which corresponds to a phylogeny after an infinitely long aging phase (i.e., a star phylogeny). Note that the γ statistic through time for the decline diversity scenario without any speciation in the decline phase falls in between the average values for the pure aging scenarios.
(TIF)
