# Supplementary figures and images for: The Molecular Phylogenetic Signature of Clades in Decline
Source: PLoS One. 2011 Oct 4;6(10):e25780. doi: 10.1371/journal.pone.0025780 (PMC3186775; doi:10.1371/journal.pone.0025780)

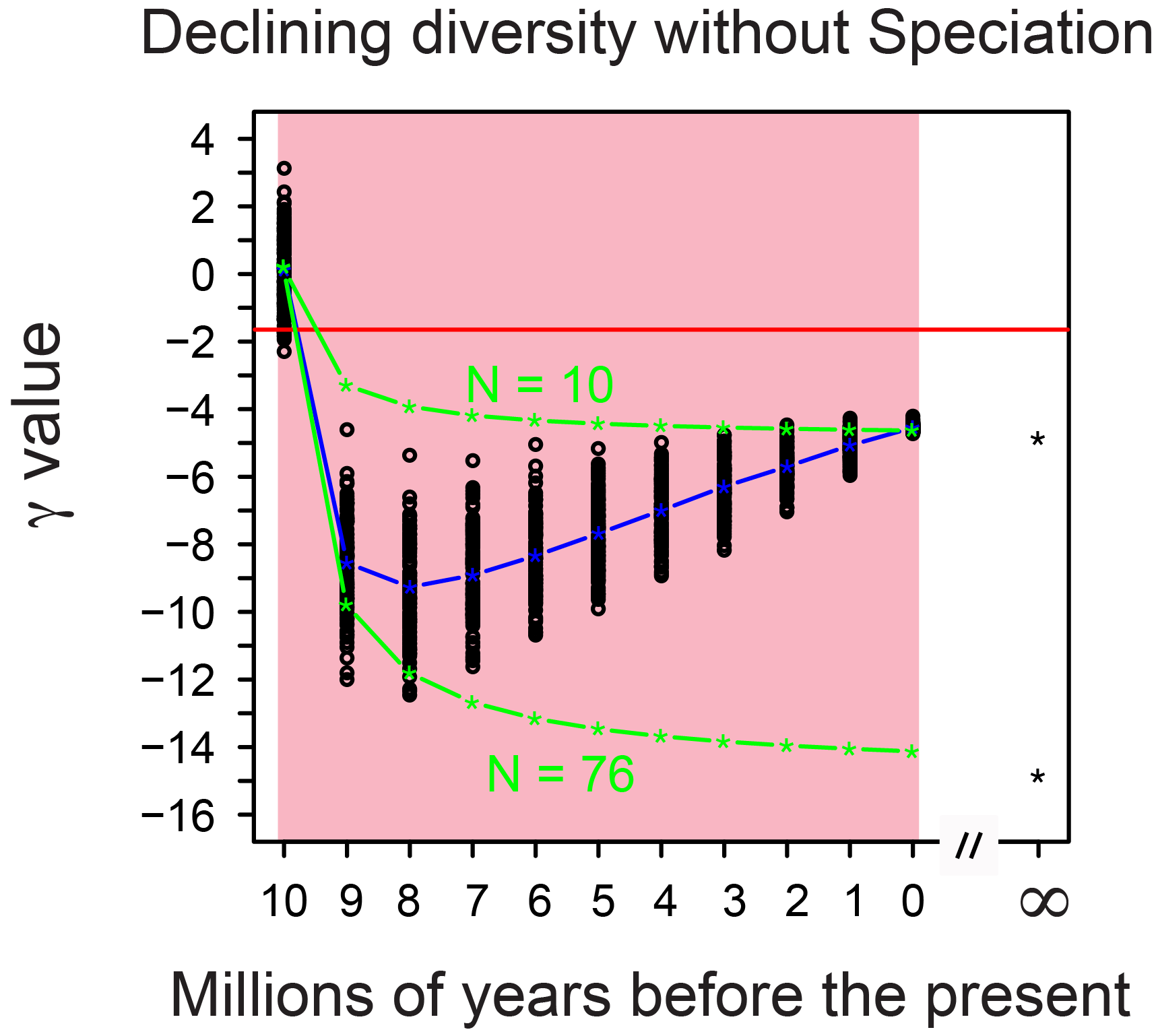

Supplement: Figure S1 — The γ statistic through time for a decline diversity scenario without any speciation in the decline phase (last 10 MY). Rates of speciation and extinction used here were chosen to produce the same diversification rates in the rise (speciation = 2.0; extinction = 0.1; r = 1.9) and decline (speciation = 0.0; extinction = 0.2; r = −0.2) phases as used in the scenario shown in figure 1B. The blue line represents the average γ statistic. The red line represents the 5% cutoff point for rejecting the null hypothesis of constant diversification (γ = −1.645). The green lines represent the average γ statistic for simulated trees in the pure aging scenario after initial exponential growth for a peak diversity of 10 and 76 species (same as in figure 5B). The asterisk at time = ∞ represents the most negative value possible for the γ statistic for a given phylogeny with 10 or 76 species, which corresponds to a phylogeny after an infinitely long aging phase (i.e., a star phylogeny). Note that the γ statistic through time for the decline diversity scenario without any speciation in the decline phase falls in between the average values for the pure aging scenarios. (TIF) [file pone.0025780.s001.tif]
